# Toward a better understanding of the uptake of interventions for cancer-related fatigue: the perspective of healthcare providers, patients, and partners

**DOI:** 10.1007/s00520-025-09765-3

**Published:** 2025-08-01

**Authors:** Kim F. Francken, Annelotte Kooij, Diana Zwahlen, Laurien M. Buffart, Joost Dekker, Hanneke W. M. van Laarhoven, Annemarie M. J. Braamse, Fabiola Müller, Hans Knoop

**Affiliations:** 1 Department of Medical Psychology, Amsterdam UMC, Location University of Amsterdam, Amsterdam, The Netherlands; 2 Cancer Center Amsterdam, Cancer Treatment and Quality of Life, Amsterdam, The Netherlands; 3 Amsterdam Public Health, Mental Health, Amsterdam, The Netherlands; 4 Medical Oncology Department, University Hospital Basel, Basel, Switzerland; 5 Department of Psychosomatic Medicine, University Hospital Basel, Basel, Switzerland; 6 Department of Medical BioSciences, Radboud University Medical Center, Nijmegen, The Netherlands; 7 Department of Psychiatry, Amsterdam UMC, Location Vrije Universiteit Amsterdam, Amsterdam, The Netherlands; 8 Department of Medical Oncology, Amsterdam UMC, Location University of Amsterdam, Amsterdam, The Netherlands

**Keywords:** Cancer, Cancer-related fatigue, Care need, Care uptake, Supportive care, Survivorship

## Abstract

**Purpose:**

While cancer-related fatigue (CRF) is prevalent among cancer patients posttreatment, the uptake of evidence-based interventions is low. This suggests resources are not well used, and patient needs go unaddressed. We investigated reasons for the discrepancy between the high prevalence of CRF and limited uptake of interventions, from the perspective of healthcare providers (HCPs), patients, and partners. We explored this from experiencing CRF, to needing care, seeking care, and use of care for CRF.

**Methods:**

Semi-structured interviews and focus groups were conducted with patients (*n* = 16) who completed curative cancer treatment and experienced CRF, partners (*n* = 11), and HCPs (*n* = 29).

**Results:**

Thematic analysis revealed five themes explaining the low uptake of care. Only patients who experienced (1) *interference due to fatigue* developed a care need. Care seeking and referral to care were hampered as it was (2) *unclear which HCP is responsible for assessment and management of posttreatment fatigue*, and because of (3) a *lack of awareness of interventions and referral pathways*, (4) *unhelpful expectations regarding fatigue and interventions*, and (5) *practical barriers influencing the initiation and continuation of care use*.

**Conclusion:**

CRF posttreatment is not routinely assessed, and it is unclear for patients and HCPs which HCP is responsible for its assessment and management. Knowledge of CRF and interventions is limited, leading to uncertainty about the treatability of CRF. To improve patients’ access to care, responsibilities of HCPs need to be defined, knowledge needs to be improved, and CRF assessment and management must be integrated into standard clinical practice.

**Supplementary Information:**

The online version contains supplementary material available at 10.1007/s00520-025-09765-3.

## Introduction

Cancer-related fatigue (CRF) is a prevalent symptom after curative treatment of cancer [1–4] and is associated with significant impairment of daily functioning [2, 5, 6]. While evidence-based interventions for CRF are recommended in international guidelines [7], that is, cognitive behavioral therapy (CBT), exercise, and mindfulness-based therapy (MBT) [7–12], few patients receive these interventions [13, 14]. A recent study conducted in the Netherlands shows that only about half of the patients who indicate a care need for CRF actually received care for this symptom [13]. Internationally, CRF is also recognized as a prominent unmet need [15–17]. This indicates that patients’ needs are not properly addressed, and healthcare resources are not well directed. An exploration of reasons for this discrepancy, where relevant factors for every step—from experiencing CRF, to having a care need, to care seeking, and care use—are identified, as well as for this pathway as a whole, is currently lacking.

Patient characteristics and psychological processes may help explain the discrepancies between experiencing a symptom and using care. Studies on distress in patients with cancer show that despite distress being present, a significant proportion of patients did not indicate a supportive care need due to a preference for self-management or limited knowledge about available care [18, 19]. Merely the presence and severity of a symptom are not sufficient to identify those who want or need care [20]. A qualitative study among patients with hematological cancer suggests that the impact of a symptom can help explain help-seeking behavior. The authors found that symptoms were more likely to result in help-seeking if they interfered with personal goals [19]. An interview study among fatigued cancer patients identified possible obstacles for care use. These include patients’ negative fatigue expectations, negative representation of interventions, and interfering physical and psychological factors (e.g., fatigue itself, pain, or fear) [21]. While providing valuable insights, these studies focused mainly on a single stage in the pathway to CRF-care.

The pathway to CRF-care cannot be seen in isolation as it is likely influenced by healthcare providers (HCPs) and a patient’s social network. While receiving a recommendation from a HCP to use psycho-oncological services can improve uptake [22], it seems that CRF is not routinely assessed by HCPs and only few patients receive advice on treatment or management [23]. These findings suggest that HCPs are not contributing optimally in the pathway to CRF-care. Furthermore, previous research does not address whether and how interactions between HCPs and patients impact the pathway to CRF-care. Additionally, previous research suggests that partners can be supportive in managing and coping with health issues [24], thus potentially influencing care need, seeking, or use. However, the role of patients’ partner’s in managing CRF has not been studied before.

Studies so far have focused on one perspective to explain the low uptake of CRF-care (either the patient or the HCP) or on isolated stages of the pathway to CRF-care. We aimed to address these limitations by investigating the entire pathway from fatigue occurrence to care need, seeking, and use while integrating the perspectives of patients, HCPs, and partners.

## Methods

### Participant selection

Participants were patients who had cancer, partners of a person who had cancer and HCPs. Patients were eligible if they (1) had cancer, (2) had received curative treatment, (3) completed treatment ≥ 6 months ago (exceptions were made for hormonal therapy), (4) had no sign of recurrence, (5) experienced moderate to severe levels of fatigue (score of ≥ 30 on the Checklist Individual Strength (CIS), subscale fatigue severity (CIS-fatigue)), and (6) were ≥ 18 years old. We also wanted to include the perspective of patients who already used care specifically for CRF. We included these patients based on their CIS-fatigue score before starting the intervention. This score was requested from their HCP with the patients’ consent. Partners of cancer patients who were treated with curative intent and experienced CRF were eligible, regardless of whether the patient was included. HCPs were eligible if they provided follow-up care for cancer survivors or if they provided evidence-based interventions for fatigue. Participants were recruited through academic hospitals, newsletters and social media of cancer support groups, another study on CRF-care in cancer patients [25], a newsletter of the Dutch Network for Physical Therapists in Oncology, and the researchers’ own networks. Purposive sampling was used to ensure a variety of participants involved in different care types (i.e., follow-up care, CBT, MBT, and physical therapy). We aimed to include a variety of HCPs, since we expected their view could be profession-specific, ranging from medical (oncologists, nurses) to generalist (GPs, social workers), psychological (psychologists), and exercise-focused (physical therapists) perspectives. Similarly, we expected views on relevant factors, barriers, and facilitators could differ among patients, depending on how far they have progressed toward care seeking or care use. Participants received information via telephone and an information letter. After obtaining informed consent, participants were screened for eligibility.

### Measures

Fatigue severity was determined with the Checklist Individual Strength (CIS), subscale fatigue severity (CIS-fatigue). The CIS consists of 20 items with a fatigue severity subscale of 8 items scored on a 7-point Likert scale (range 8–56). We applied a cut-off score of ≥ 30 for moderate fatigue which was calculated as 0.5SD above the mean CIS-fatigue score from a population-based sample [26]. Information on diagnosis and treatment were collected from medical records. Partners of patients, the latter experiencing CRF as reported by the patient or the partner if it did not concern an included couple, filled out a demographic questionnaire. Screening of HCPs consisted of a questionnaire regarding occupational information and an eligibility check via e-mail.

### Participant interviews

Semi-structured interviews and focus groups were conducted either face-to-face or online through a secure video connection. Focus groups consisted of a maximum of 5 individuals, grouped per profession. If attending a focus group was not feasible, HCPs were interviewed individually. We followed a semi-structured guide (see Appendix 1) that was developed by the research team, consisting of experienced researchers in the field of psycho-oncology and dyadic coping, clinical psychologists experienced with CBT for CRF, and a medical oncologist. Patient interviews focused on patients’ needs for supportive care, their experiences with care seeking and use, the role of HCPs, and the involvement of partners. Partners were asked about their perceptions of patients’ fatigue, care need, seeking, and use. HCPs were asked to share their perspectives on care options for CRF and their experience in discussing CRF with patients and referrals to supportive care interventions. The interviewers (KF and AK) did not have any type of relationship with the participants.

### Data analysis

Interviews and focus groups were audio recorded, transcribed, and analyzed with MaxQDA software (version 2022 Plus), using a thematic approach entailing multiple steps: coding, generating themes, reviewing themes, and defining themes [27]. Two researchers independently coded the interviews (KF and AK, KF and FM). Data saturation was determined by observing code emergence across interviews and was reached when no new relevant codes emerged. Themes were identified separately by two researchers (KF and FM); the research questions and the different perspectives of HCPs, patients, and partners guided this process. Themes were discussed and redefined; consensus was reached through discussions and consulting HK. Participants were provided a summary of the results and were given the opportunity to provide feedback on the interpretation and themes. This did not lead to revisions of the results.

## Results

### Sample characteristics

We screened 23 patients, 15 partners, and 32 HCPs for eligibility. Sixteen patients, 11 partners, and 29 HCPs were eligible. Main reasons for ineligibility were (1) disease recurrence, (2) not able to find time for the interview or focus group, and (3) unable to reach the participant after initial contact. We conducted 6 focus groups with 3 to 5 HCPs and 9 individual interviews with HCPs. Individual interviews lasted 40–60 min, and focus groups lasted 60–90 min. See Tables 1 and 2 for characteristics of participants.

**Table 1:** Sample characteristic patients and partners

Sample characteristics	Patients (*N* = 16)	Partners (*N* = 11)
Sex (female), *n* (%)	13 (81.2%)	4 (36.4%)
Age in years, median (range)	54.0 (36–81)	59.0 (46–69)
Relationship status^b^ (has a partner), *n* (%)	11 (68.8%)	11 (100.0%)
Education^c^, *n* (%)		
Low/medium	7 (43.8%)	5 (45.5%)
High	9 (56.3%)	6 (54.5%)
Occupational status^**a**^, *n* (%)		
Paid work	5 (31.3%)	7 (63.6%)
Retired	3 (18.8%)	2 (18.2%)
Unpaid work	0 (0.0%)	1 (9.1%)
Not able to work	9 (56.3%)	1 (9.1%)
Others	0 (0.0%)	1 (9.1%)
Diagnosis, *n* (%)		
Breast cancer	10 (62.5%)	n.a.
Other (cervical, ovarian, skin, intestinal, lymphatic, bone marrow)	6 (37.5%)	n.a.
Time since end of tx in months, median (range)^d^	45 (11–115)	n.a.
CIS-fatigue score, median (range)	50.0 (33–56)	n.a.
Supportive care use posttreatment^**a**^, *n* (%)		
CBT/psychological care for CRF	5 (31.3%)	n.a.
CBT/psychological care for other symptom	10 (62.5%)	n.a.
Physical therapy (not specifically for CRF)	13 (81.2%)	n.a.
MBT for CRF	1 (6.3%)	n.a.
MBT for other symptom	1 (6.3%)	n.a.
Oncological rehabilitation program (not specifically for CRF)	4 (25.0%)	n.a.

*tx*, treatment; *CIS-fatigue*, Checklist Individual Strength, fatigue subscale; *CBT*, cognitive behavioral therapy; *CRF*, cancer-related fatigue; *MBT*, mindfulness based therapy; ^a^counts up to more than the total sample size because participants could select multiple answers; ^b^6 couples were included; ^c^low/medium = primary school, lower secondary education, medium secondary education, high = higher secondary education, higher professional education, University; ^d^time since treatment was calculated based on the last date of the last treatment a patient received, until the date of inclusion in our study, hormonal therapy could be ongoing and was not considered in determining time since treatment

**Table 2:** Sample characteristics HCPs

Sample characteristics	Healthcare providers (*N* = 29)
Sex (female), *n* (%)	22 (75.8%)
Age in years, median (range)	46.5 (26–66)
Profession, *n* (%)	
Oncologist (internal, radiotherapy, rehabilitation, gynecological, hematological)	6 (20.7%)
Nurse (specialist)	4 (13.8%)
General practitioner	4 (13.8%)
Physical therapist	5 (17.2%)
Psychologist	5 (17.2%)
Medical social work	5 (17.2%)
Setting^a^, *n* (%)	
University Medical Center	21 (72.4%)
Physical therapy practice	4 (13.8%)
General practitioner practice	4 (13.8%)
Other (healthcare center, mental healthcare institution)	2 (13.8%)
Years working in oncology, mean (range)	15 (0.5–38)

^a^Counts up to more than the total sample size because participants could indicate multiple answers

### The process from experiencing fatigue to care use

**Fig. 1:**
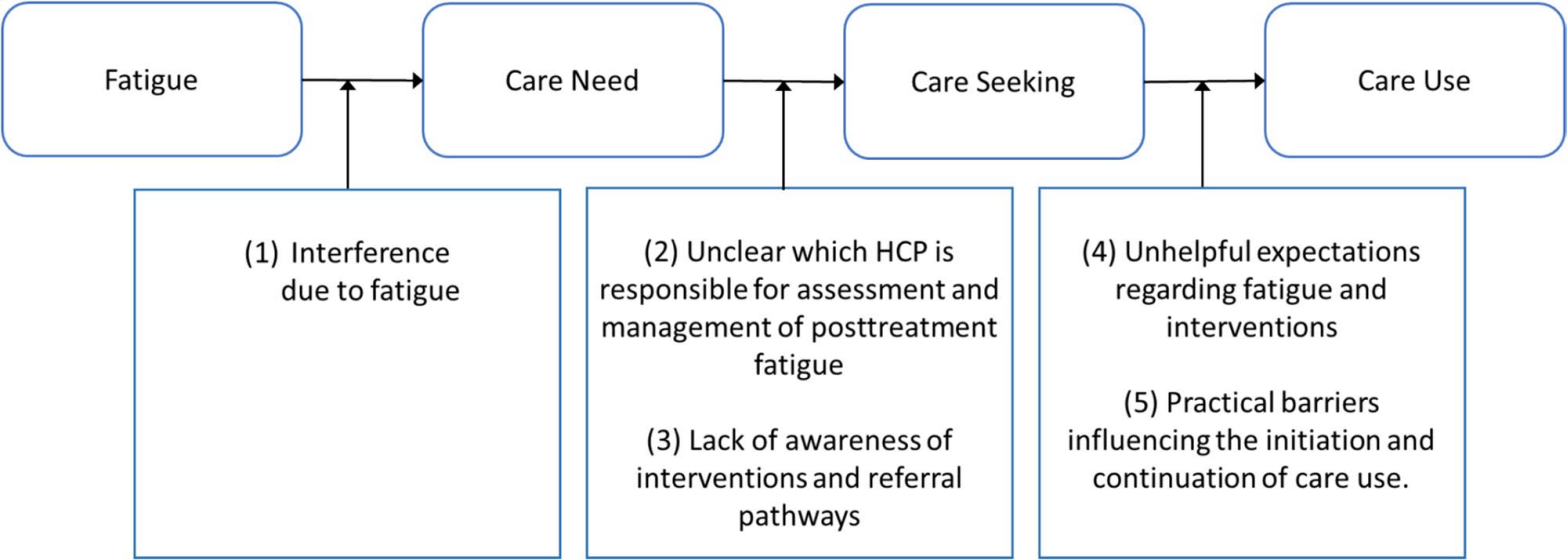
Schematic and simplified outline of the process from experiencing posttreatment fatigue to care use and identified themes

The interviews yielded 5 primary themes: (1) interference due to fatigue, (2) unclear which HCP is responsible for assessment and management of posttreatment fatigue, (3) lack of awareness of interventions and referral pathways, (4) unhelpful expectations regarding fatigue and interventions, and (5) practical barriers influencing the initiation and continuation of care use. Figure 1 illustrates how the themes relate to each step of the care use process. Quotes for all themes are presented in Table 3.

**Table 3:** Exemplary participant quotes presented per theme

	Healthcare providers	Patients	Partners
(1) Interference due to fatigue		“And now, I can better accept it. If I want to do something, I plan it in the morning, do it for an hour, if I get tired I stop. […] I found the balance to deal with it I think.” [F4, 57y, breast cancer] “Yes. That I really thought, if this is it then I really have a problem. If this doesn’t get better, I mean. If I keep having this fatigue, then that really is a problem.” [F1, 52y, cervical cancer]	“But it was more from herself, the wish and the desire [for supportive care]. She absolutely could not accept that this was it, so to speak, that this was the maximum that could be achieved. She was like, ‘There has to be more’.” [M2, 46y] “I support her in everything. So that- But she has to do it herself.” [M5, 69y]
(2) Unclear which HCP is responsible for assessment and management of posttreatment fatigue	“You need to make a distinction between the long-term fatigue, posttreatment (fatigue) or during treatment. Because during treatment, we find it extremely important because patients will not get through their treatment, so we really emphasize it then. […] After [cancer]treatment, we are less pushy. […] if we don’t have any tools… only our words… […]. You already mentioned it, gave some advice, but we can’t offer more than that. You feel powerless.” [M2, 53, oncologist] “And I wonder to what extent patients also see a role for the GP in this. I think that that role is present, mainly because we are so generalistic, because we have a holistic approach. But I think that patients themselves will also make a connection between, for example, the treatment or the diagnosis and the fatigue that follows, and therefore first return to the specialist with that question.” [M6, 31y, general practitioner]	“I am and remain searching, if there would be a checklist somewhere, what you can do about fatigue, I would like to have it.” [F7, 59y, breast cancer] “Like, who should I go to, who should I go to—should I go to a psychologist, or should I go to a coach? Or should I go do exercise?” [F8, 54y, breast cancer]	
(3) Lack of awareness of interventions and referral pathways	“Give me tools, I would love to refer” [M1, 66y, oncologist] “We don’t ask about it [fatigue] if we cannot do anything about it” [M2, 53y, oncologist] “Yes, I do refer a lot of patients to oncological rehabilitation program. Honestly, I just wing it, I have no idea.” [F15, 43y, oncologist] “Because I have always said about that: it is a very strange Russian roulette for the patients. That it really depends on so many factors that determine whether they are offered something for it or not, while yes, it does not mean that if someone does not show it a lot, that it is not there. And that someone who is doing quite well in their daily lives, that they could still actually use some help with that. But yeah, if they do not say it themselves and it does not come up as a kind of issue during aftercare sessions with the doctor or something, yeah, then they will not find me.” [F18, 31y, social worker]	“It's strange that I don’t know this [interventions for CRF], right!?” [F11, 81y, esophageal cancer] “I didn’t search for it anymore either. Also because the oncologist told me there wasn’t anything for it [CRF].” [M1, 54y, lympfatic cancer]	
(4) Unhelpful expectations regarding fatigue and interventions	“When you finished treatments, it makes sense you are tired, it has been normalized. […]. When they talk about it [in the hospital] it gets dismissed a bit, ‘it will pass’ or ‘learn to live with it’. […] I think they need help with this early on, that it should be better defined, if they are tired the first half year it’s okay but it should get better, and if not, let us know. You want that to be the message from the hospital. […] But that is not the message they receive and I think that is where it goes wrong. Despite having received some education [about fatigue], to my shock, there are still oncologists who say yes, you have to learn to live with it. That still happens”. [F3, 50y, CBT psychologst] “Yes, and ignorance of… well, a bit the old view of cracking, massaging, those type of things. […] Yes, and if you- if that’s your frame of reference, then you see people don’t come to you.” [M4, 52y, physical therapist]	“He [the oncologist] said, yes [name of participant], it will not change, you have to – you’re going to have to deal with the fact that you just have this energy level. And that’s not going to change that much.” [F12, 46y, breast cancer] “And it’s not that I don’t have a care need, but such an intensive process [CBT], makes me think: that just doesn’t fit within my life. Only if there was something that maybe, that was easy to follow or something that you could do remotely or, I don’t know, just something like that.” [F6, 36y, breast cancer] “And whether it is psychological, in your head, I find it unlikely. I think, […] something just changed in my DNA.” [M1, 54y, lymphatic cancer] “The point is […] it is a generic treatment, and I noticed that it’s becoming a more important aspect [for me] that I had cancer. That is increasingly playing a role. So now I think, yes, I would actually like to have conversations with a psychologist who specializes in cancer, in fear after cancer and fatigue after cancer.” [F5, 57y, ovarian cancer]	
(5) Practical barriers influencing the initiation and continuation of care use		“I still remember that I went to enroll for CBT and that I read on the website that it could take 9 to 12 months. That was already such a big mountain for me that I thought, never mind. And he [partner] said no, this is a gift, you really must take this, it really needs time. And when I was really down or frustrated or felt like it wasn’t going fast enough, he always brought me the peace of mind, like no, you’re going to continue, you’ve come this far.” [F10, 45y, breast cancer] “So I wanted to do Oncofit [a cancer specific exercise program]. That’s one of those programs. I had just started, because they had said that there was two years of insurance. Because yes, I can’t afford it, the exercise. So I, yeah, I’m on sick leave now. I just have a social assistance income, so it’s just too expensive. I just started, I was really enjoying it, and then they said oh, a notification from the insurance, you only get one year insured.” [F5, 57y, ovarian cancer]	“I said: go to a psychologist. I said: those are people who have studied for years, right, they know exactly the right points, they are not emotionally close to you, because if you say: yes, but I can tell a girlfriend. I say: no, then you are emotionally involved and then you get very different guidance, than if you have someone next to you who- Ehh, who knows how to guide you in- How should I say this, professional ways.” [F1, 54y] “Yes, and related to that: you know, what does it mean for our income? What does it mean for our finances? She really worries about that, I worry less.” [M1, 59y] “Yes. Yes, less straining, right, try to ensure less burden but also invite fewer people over, you know, fewer activities. We do those kinds of things. But that’s not the prevention- It’s not solving the fatigue, those are things you do because there is fatigue.” [M6, 55y]

*F*, female;* M*, male;* y*, years of age; *CRF*, cancer-related fatigue

### From fatigue to care need

#### Interference due to fatigue

Patients indicated that, after cancer treatment, they aimed to resume their lives as before diagnosis and treatment. However, if, unexpectedly, fatigue persisted and hindered this return, this was identified as a “turning point”. While some patients accepted this new situation and adjusted their lives accordingly (e.g., working less hours), others found it difficult to accept the impact of fatigue and developed a care need. Partners confirmed that the patient’s fatigue led to either acceptance or frustration, the latter often leading to a care need. Partners described the patients’ desire to resume valued activities as an important driver. Despite fatigue being described as impactful and requiring adjustments from the partner as well, partners stressed they tried to be supportive in patients’ necessary adjustments due to fatigue, such as helping manage social activities or doing household chores.

### From care need to care seeking

#### Unclear which HCP is responsible for assessment and management of posttreatment fatigue

A contrast was noted by oncologists regarding fatigue during cancer treatment compared to after treatment completion. Several oncologists explained that addressing fatigue during cancer treatment can be crucial for treatment continuation and therefore is their responsibility. However, after treatment, the urgency to address fatigue diminished and most felt uncertain how to address and manage fatigue. Patients however described how fatigue became increasingly important if it persisted after treatment. Nearly all patients indicated they were unsure where to seek care if fatigue became a persistent complaint. It is unclear for both patients and HCPs who is responsible for the management of persistent CRF. They described that there is no designated HCP for the evaluation or management of fatigue posttreatment. All oncologists acknowledged that fatigue after cancer is an important issue, but most felt “empty handed.” Although they sometimes asked patients about their physical condition and fitness, discussing fatigue is not part of standard clinical practice. Furthermore, patients and oncologists described that the focus of follow-up consultations was usually on the medical aspect (i.e., scan results) which was often in line with the priority of the patient. Oncologists indicated they often only discussed fatigue when it was mentioned by patients. However, patients indicated they would prefer that an oncologist or nurse would take the initiative to address fatigue and offer help because they did not want to burden their oncologist within the already busy healthcare system. Patients, oncologists, and nurses indicated nurses could potentially be the designated HCP to discuss and manage fatigue posttreatment.

General practitioners (GPs) reported that patients rarely come to them with fatigue complaints; they assumed patients do not see them as the right HCP to seek care for cancer-related symptoms. GPs reported a lack of knowledge on how to assess and manage fatigue was a barrier for providing advice on CRF. One GP suggested there could be a useful role for GPs in a patients’ care seeking process, as they could offer a holistic approach to the assessment of fatigue.

#### Lack of awareness of interventions and referral pathways

Several patients, oncologists, nurses, and GPs reported they were unaware of interventions for posttreatment fatigue. Despite having a care need, some patients indicated they did not seek care because they did not know that interventions for fatigue exist. Similarly, oncologists mentioned they did not know what to do about posttreatment fatigue and did not have any tools to assess and discuss it. Most oncologists were unaware of (international) guidelines for CRF. Oncologists and nurses mentioned they did not have the tools to assess fatigue, but some used patient-reported outcome measures to identify symptoms patients were struggling with, which sometimes led to self-management advice and occasionally referral to physical therapy or a psychologist. Those who were aware of (some) existing interventions indicated fatigue is often a multifactorial symptom, and without clear guidance, they did not know which patient could benefit from which intervention. Patients explained it was often due to their own assertiveness or “just coincidence” whether they actually found and received care, and there was no clear pathway to initiate seeking care. For example, patients reported that they just happened to know a physical therapist or coincidentally a friend recommended an intervention.

### From care seeking to care use

#### Unhelpful expectations regarding fatigue and interventions

Patients’ and HCPs’ expectations regarding the treatability of fatigue and effectiveness of interventions appeared to influence patients’ care use. Patients tended to align with their oncologists’ beliefs on treatability. If their HCP labeled fatigue as normal, most patients accepted this statement. Additionally, due to some patients’ own fatigue attributions, they did not expect that a certain intervention would benefit them and thus did not use it. For example, several patients believed their fatigue was due to physical damage from cancer or treatment and, therefore, did not expect a psychological intervention would be effective in reducing fatigue. Patients also mentioned they prefer interventions delivered by HCPs with specific CRF expertise. For example, one patient felt the psychological intervention she followed was not effective, as it was too generic. She did expect she could benefit from a psychological intervention if it was more cancer- or fatigue-specific and delivered by a HCP specialized in CRF. Additionally, patients’ expectations of intervention suitability and the possibility to integrate it into their daily life seemed to be essential. For example, some patients indicated an intensive CBT intervention was impossible to integrate into their busy family life. Lastly, some did not expect professional physical therapy to help as they already tried increasing physical activity themselves, without the expected improvement.

Psychologists and physical therapists noticed negative preconceptions among patients and other HCPs (e.g., psychological interventions are not suitable for fatigue, physical therapy is for muscle or injury treatment, or mindfulness is “just meditating”), which they expected could hinder referral. Psychologists explained that often the referring HCPs are unaware of the existence or benefits of CBT or MBT for fatigue or do not know how or when to refer to it. Furthermore, they assumed that when oncologists or nurses had the perception that “fatigue is normal” after having had cancer, this could hinder referral. Physical therapists had more positive experiences regarding the number of patients that were referred to physical therapy, as they were often already involved from the start of diagnosis and during treatment. However, patients were rarely referred specifically for fatigue.

#### Practical barriers influencing the initiation and continuation of care use

Several patients reported that insufficient insurance coverage and, occasionally, travel distance hindered care use. Waiting lists for psychological care could be a barrier and were mainly seen as a frustration. However, some patients did enroll, despite long waiting lists. In some cases, partners encouraged the patient to enroll. Some patients described that their partner supported them in practical ways, relieving some of the barriers (e.g., by providing income and taking over household chores) to enable them to follow CRF-care. Physical therapists described patients often drop out when insurance coverage ends. Psychologists noticed frustration with patients regarding the waiting lists.

## Discussion

This study suggests that several factors underlie the discrepancy between the high prevalence of CRF and the limited uptake of supportive care interventions. A care need for fatigue occurs in patients who are unable to resume valued activities due to fatigue and are unwilling to accept this. Care seeking is challenging for patients, as there is no clear path to seek CRF-care. It is unclear for patients and also for HCPs which HCP is responsible for the assessment and management of CRF. HCPs are often either unaware of interventions or they are unsure which intervention suits which patient. Care use is often hindered by preconceptions regarding the treatability of fatigue and (efficacy and suitability of) available interventions. Consequently, progressing to care use now mainly depends on patients’ assertiveness or on coincidence.

Our study suggests that merely screening for the severity of a symptom is not sufficient to identify patients with a care need. We find, as has been found previously for fatigue [28–30], that a subgroup of patients respond to CRF by adjusting their lives to the limitations they experience and, hence, may not develop a care need. Our study shows patients who experience interference of fatigue in their daily lives develop a care need. We also showed that even when a care need exists, the complex interplay of several factors at the patient and healthcare system level hampered seeking and using care.

The key finding of our study is that there is no clear pathway to posttreatment CRF-care in the Netherlands, and responsibilities among HCPs are undefined. It is unclear who is responsible for addressing fatigue posttreatment, and patients do not know where to seek care. We found several explanations for this. As found previously [23, 30–33], many of the interviewed HCPs indicated they had not heard of guidelines for CRF and did not have a systematic approach for assessing and managing CRF posttreatment. While limited knowledge and uptake of clinical guidelines is a challenge in CRF-care [31] and in healthcare in general [34–37], our study revealed a potential explanation for this challenge at the HCP level. That is, we observed a divergence between HCPs and patients regarding the priority of fatigue. While HCPs see addressing fatigue during cancer treatment as their responsibility and as a priority and have the tools to address fatigue (e.g., lower treatment dosage), this fades over time when treatment is completed and medical causes are less clear. However, for patients, survival is a priority during treatment, and fatigue feels less relevant. Fatigue becomes more of a priority after treatment has been completed and when CRF persists and interferes with their daily lives. Since the advice from a HCP can influence the uptake of care [22], and patients in our study also indicated they prefer to follow the advice from their HCP, the active role of HCPs is essential in overcoming the discrepancy between the high prevalence of CRF and the limited uptake of care. This finding stresses the importance of defining responsibilities regarding CRF-care.

### Clinical implications

Identifying patients with a care need for CRF and addressing this need properly requires an integrative approach. Merely screening for symptom severity is not sufficient. Our findings suggest it requires a system that takes into account the challenges of the multifactorial character of fatigue, its development over time, and the different factors underlying care need, seeking, and use. Enhancing HCPs’ understanding of posttreatment CRF (e.g., by integrating posttreatment CRF into education or advocate guidelines through professional associations) can empower them to provide appropriate advice after treatment and overcome misperceptions regarding the treatability of and interventions for CRF. While we recognize merely increasing knowledge is not enough, it can be an important contributor to change. Our findings do not provide insight on how to ensure that knowledge is implemented in daily practice; however, previous research suggests several strategies: knowledge should be made easily accessible to busy HCPs, processes should be time efficient, and they should align well with existing practice [32].

Mainly, responsibilities need to be defined. We suggest a system that combines empowering patients with the expertise of a trained HCP. Acknowledging that patient preferences differ, information about potential long-term fatigue and interventions should be provided during and after treatment, e.g., through leaflets, campaigns, existing platforms, or self-help apps. This could encourage access to self-management strategies and knowledge about the value of available interventions. When responsibilities regarding management and assessment of CRF are defined, patients’ care needs can be timely identified. A specialized nurse has the potential to fulfill this responsibility. To enable this role of the nurse within the current healthcare system, specific nurse training might be needed to increase knowledge of guidelines and interventions and self-efficacy in counseling patients regarding CRF [38]. Additionally, previous research has shown that the early introduction of a trained nurse counselor for CRF can increase a patients’ ability to engage in supportive care discussions [39]. This nurse could identify the patients’ needs, aid patients’ self-management of fatigue, provide basic fatigue care, or guide the patient to find suitable care. Ideally, supported by HCPs that deliver evidence-based CRF-care, e.g., a psychologist [40] or a physical therapist. This combination could improve the pathway to fatigue interventions, without putting too much additional strain on an already burdened healthcare system.

### Limitations

As most HCPs were recruited from one university medical center, our results may not reflect other settings in the Netherlands. Relatedly, the structure of the Dutch healthcare system might limit generalizability to other healthcare systems due to a separation of responsibilities between GPs and supportive care (primary care), non-academic hospitals (secondary care), and academic hospitals (tertiary care). However, some findings related to challenges at the healthcare system level are evidently also at play in other countries [30–32], suggesting they are relevant across various healthcare systems. Some participants were recruited through an intervention study for fatigue, and some of the interviewed HCPs were familiar with this study. This could have limited the generalizability to other settings due to an overrepresentation of knowledge about CRF interventions in this group. Lastly, the study was conducted in the Netherlands, with mostly white female participants and mainly in an academic setting, potentially limiting the generalizability of our findings.

## Conclusion

In our study, conducted in the Netherlands, CRF after treatment appeared not to get routinely assessed by HCPs—both in the hospital setting or general practice, and it is unclear which HCP is responsible for its assessment and management. Knowledge of fatigue and evidence-based interventions is limited in patients and HCPs, leading to uncertainty about the treatability of CRF. To increase the uptake of evidence-based interventions and properly identify and address patients’ needs, responsibilities among HCPs regarding CRF need to be defined. Patients need to be empowered with knowledge and self-management tools to address fatigue and, when needed, be referred to evidence-based interventions.

## Supplementary Information

Below is the link to the electronic supplementary material.

Supplementary file1 (DOCX 31.5 KB)

## Data Availability

The data generated during and/or analyzed during the current study are available from the corresponding author on reasonable request.

## References

[1] Susanne K, Michael F, Thomas S, Peter E, Andreas H (2019) Predictors of fatigue in cancer patients: a longitudinal study. Support Care Cancer 27(9):3463–3471. 10.1007/s00520-019-4660-4 30680616 10.1007/s00520-019-4660-4

[2] Jones JM, Olson K, Catton P, Catton CN, Fleshner NE, Krzyzanowska MK et al (2016) Cancer-related fatigue and associated disability in post-treatment cancer survivors. J Cancer Surviv 10(1):51–61. 10.1007/s11764-015-0450-2 25876557 10.1007/s11764-015-0450-2

[3] Bower JE, Ganz PA, Desmond KA, Bernaards C, Rowland JH, Meyerowitz BE et al (2006) Fatigue in long-term breast carcinoma survivors - a longitudinal investigation. Cancer 106(4):751–758. 10.1002/cncr.21671 16400678 10.1002/cncr.21671

[4] Kang YE, Yoon JH, Park NH, Ahn YC, Lee EJ, Son CG (2023) Prevalence of cancer-related fatigue based on severity: a systematic review and meta-analysis. Sci Rep 13(1):12815. 10.1038/s41598-023-39046-0 37550326 10.1038/s41598-023-39046-0 PMC10406927

[5] Abrahams HJG, Gielissen MFM, Verhagen CAHHVM, Knoop H. (2018) The relationship of fatigue in breast cancer survivors with quality of life and factors to address in psychological interventions: a systematic review. Clin Psychol Rev. 63:1–11 10.1016/j.cpr.2018.05.004 10.1016/j.cpr.2018.05.004 29852324

[6] Müller F, Tuinman MA, Janse M, Almansa J, Sprangers MAG, Smink A et al (2017) Clinically distinct trajectories of fatigue and their longitudinal relationship with the disturbance of personal goals following a cancer diagnosis. Brit J Health Psych 22(3):627–643. 10.1111/bjhp.12253 10.1111/bjhp.12253 28635083

[7] Bower JE, Lacchetti C, Alici Y, Barton DL, Bruner D, Canin BE et al (2024) Management of fatigue in adult survivors of cancer: ASCO-Society for Integrative Oncology guideline update. J Clin Oncol 42(20):2456–2487. 10.1200/JCO.24.00541 38754041 10.1200/JCO.24.00541 PMC12082589

[8] Mustian KM, Alfano CM, Heckler C, Kleckner AS, Kleckner IR, Leach CR et al (2017) Comparison of pharmaceutical psychological and exercise treatments for cancer-related fatigue a meta-analysis. Jama Oncol 3(7):961–968. 10.1001/jamaoncol.2016.6914 28253393 10.1001/jamaoncol.2016.6914 PMC5557289

[9] van der Lee ML, Garssen B (2012) Mindfulness-based cognitive therapy reduces chronic cancer-related fatigue: a treatment study. Psychooncology 21(3):264–272. 10.1002/pon.1890 22383268 10.1002/pon.1890

[10] Witlox L, Hiensch AE, Velthuis MJ, Bisschop CNS, Los M, Erdkamp FLG, et al. 2018 Four-year effects of exercise on fatigue and physical activity in patients with cancer. Bmc Med. 16 10.1186/s12916-018-1075-x. 10.1186/s12916-018-1075-x PMC5992660 29879968

[11] Corbett TK, Groarke A, Devane D, Carr E, Walsh JC, McGuire BE. 2019 The effectiveness of psychological interventions for fatigue in cancer survivors: systematic review of randomised controlled trials. Syst Rev-London. 8(1) 10.1186/s13643-019-1230-2 10.1186/s13643-019-1230-2 PMC6911282 31836007

[12] Abrahams HJG, Gielissen MFM, Donders RRT, Goedendorp MM, van der Wouw AJ, Verhagen CAHHVM, et al. (2017) The efficacy of internet-based cognitive behavioral therapy for severely fatigued survivors of breast cancer compared with care as usual: a randomized controlled trial. Cancer. 123(19):3825–34 10.1002/cncr.30815 10.1002/cncr.30815 28621820

[13] Engelen VM, R; Schrieks, M. (2021) Sociale steun begrip en nazorg bij kanker: wat is jouw ervaring? NFK

[14] van Willigen NE, V. (2024) Verderleven met of na kanker, hoe is dat voor jou? NFK

[15] Siao CL, Chang WC, Chen CH, Lee YH, Lai YH (2024) Symptoms, distress finances social support resource utilization and unmet care needs of patients with gynecological cancer. Eur J Oncol Nurs 72:102686. 10.1016/j.ejon.2024.102686 39317144 10.1016/j.ejon.2024.102686

[16] Hall A, Campbell HS, Sanson-Fisher R, Lynagh M, D’Este C, Burkhalter R et al (2013) Unmet needs of Australian and Canadian haematological cancer survivors: a cross-sectional international comparative study. Psychooncology 22(9):2032–2038. 10.1002/pon.3247 23436539 10.1002/pon.3247

[17] Di Meglio A, Charles C, Martin E, Havas J, Gbenou A, Flaysakier JD, et al. (2022) Uptake of recommendations for posttreatment cancer-related fatigue among breast cancer survivors. J Natl Compr Canc Netw. 20(13) 10.6004/jnccn.2021.7051 10.6004/jnccn.2021.7051 35130491

[18] Clover KA, Mitchell AJ, Britton B, Carter G (2015) Why do oncology outpatients who report emotional distress decline help? Psychooncology 24(7):812–818. 10.1002/pon.3729 25504987 10.1002/pon.3729

[19] Braamse AMJ, van Meijel B, Visser OJ, Huijgens PC, Beekman ATF, Dekker J. (2017) Help-seeking behaviour of patients with haematological malignancies treated with autologous stem cell transplantation. Eur J Cancer Care. 26(5) 10.1111/ecc.12454 10.1111/ecc.12454 26840911

[20] Dekker J, Braamse A, Schuurhuizen C, Beekman ATF, van Linde M, Sprangers MAG et al (2017) Distress in patients with cancer - on the need to distinguish between adaptive and maladaptive emotional responses. Acta Oncol 56(7):1026–1029. 10.1080/0284186x.2017.1280848 28145789 10.1080/0284186X.2017.1280848

[21] Charles C, Di Meglio A, Arnedos M, Arvis J, Baciarello G, Blanchard P et al (2021) QualFatigue study: which factors influence the use of specific interventions for breast cancer survivors with fatigue? A cross-sectional exploratory study. Support Care Cancer 29(8):4827–4834. 10.1007/s00520-021-06040-z 33547524 10.1007/s00520-021-06040-z

[22] Nascimento AF, Tondorf T, Rothschild SI, Koller MT, Rochlitz C, Kiss A et al (2019) Oncologist recommendation matters!Predictors of psycho-oncological service uptake in oncology outpatients. Psychooncology 28(2):351–357. 10.1002/pon.4948 30466146 10.1002/pon.4948

[23] Ripamonti CI, Antonuzzo A, Bossi P, Cavalieri S, Roila F, Fatigoni S (2018) Fatigue, a major still underestimated issue. Curr Opin Oncol 30(4):219–225. 10.1097/CCO.0000000000000451 29877886 10.1097/CCO.0000000000000451

[24] Gil N, Fisher A, Beeken RJ, Pini S, Miller N, Buck C et al (2022) The role of partner support for health behaviours in people living with and beyond cancer: a qualitative study. Psychooncology 31(11):1997–2006. 10.1002/pon.6032 36097392 10.1002/pon.6032 PMC9828063

[25] Harnas SJ, Knoop H, Evertsz FB, Booij SH, Dekker J, van Laarhoven HWM, et al. (2021) Personalized versus standard cognitive behavioral therapy for fear of cancer recurrence, depressive symptoms or cancer-related fatigue in cancer survivors: study protocol of a randomized controlled trial (MATCH-study). Trials. 22(1) 10.1186/s13063-021-05657-z 10.1186/s13063-021-05657-z PMC8507219 34641961

[26] Braamse A, Voss H, Nikolaus S, Wearden A, Knoop H (2020) The role of partners’ fatigue and the patient-partner relationship in the outcome of cognitive behavioural therapy for chronic fatigue syndrome. J Psychosom Res 135:110133. 10.1016/j.jpsychores.2020.110133 32450339 10.1016/j.jpsychores.2020.110133

[27] Braun V, Clarke V (2006) Using thematic analysis in psychology. Qual Res Psychol 3(2):77–101. 10.1191/1478088706qp063oa

[28] Bootsma TI, Schellekens MPJ, van Woezik RAM, Slatman J, van der Lee ML (2021) Forming new habits in the face of chronic cancer-related fatigue: an interpretative phenomenological study. Support Care Cancer 29(11):6651–6659. 10.1007/s00520-021-06252-3 33954822 10.1007/s00520-021-06252-3 PMC8464573

[29] Bootsma TI, Schellekens MPJ, van Woezik RAM, van der Lee ML, Slatman J (2020) Experiencing and responding to chronic cancer-related fatigue: a meta-ethnography of qualitative research. Psychooncology 29(2):241–250. 10.1002/pon.5213 31442340 10.1002/pon.5213 PMC7027742

[30] Campbell R, Shaw JM, Carlick T, Banks H, Faris MM, Jeon MS et al (2024) Such a different type of tiredness: people with brain tumour, their caregivers’, and healthcare professionals’ qualitative perceptions of cancer-related fatigue. J Cancer Surviv. 10.1007/s11764-024-01691-3 39405030 10.1007/s11764-024-01691-3 PMC13144180

[31] Jones G, Gollish M, Trudel G, Rutkowski N, Brunet J, Lebel S (2021) A perfect storm and patient-provider breakdown in communication: two mechanisms underlying practice gaps in cancer-related fatigue guidelines implementation. Support Care Cancer 29(4):1873–1881. 10.1007/s00520-020-05676-7 32793998 10.1007/s00520-020-05676-7

[32] Pearson EJ, Denehy L, Edbrooke L. (2023) Identifying strategies for implementing a clinical guideline for cancer-related fatigue: a qualitative study. Bmc Health Serv Res. 23(1) 10.1186/s12913-023-09377-9 10.1186/s12913-023-09377-9 PMC10127293 37095506

[33] Martin E, Zingarello A, Di Meglio A, Baciarello G, Matias M, Charles C et al (2021) A qualitative evaluation of the use of interventions to treat fatigue among cancer survivors: a healthcare provider’s view. Eur J Cancer Care (Engl) 30(2):e13370. 10.1111/ecc.13370 33191520 10.1111/ecc.13370

[34] Correa VC, Lugo-Agudelo LH, Aguirre-Acevedo DC, Contreras JAP, Borrero AMP, Patino-Lugo DF et al (2020) Individual, health system, and contextual barriers and facilitators for the implementation of clinical practice guidelines: a systematic metareview. Health Res Policy Syst 18(1):74. 10.1186/s12961-020-00588-8 32600417 10.1186/s12961-020-00588-8 PMC7322919

[35] Lugtenberg M, Zegers-van Schaick JM, Westert GP, Burgers JS (2009) Why don’t physicians adhere to guideline recommendations in practice? An analysis of barriers among Dutch general practitioners. Implement Sci 4:54. 10.1186/1748-5908-4-54 19674440 10.1186/1748-5908-4-54 PMC2734568

[36] Weller FS, Hamming JF, Repping S, van Bodegom-Vos L. (2023) What information sources do Dutch medical specialists use in medical decision-making: a qualitative interview study. Bmj Open. 13(10) 10.1136/bmjopen-2023-073905 10.1136/bmjopen-2023-073905 PMC10565272 37798031

[37] Francke AL, Smit MC, de Veer AJE, Mistiaen P. (2008) Factors influencing the implementation of clinical guidelines for health care professionals: a systematic meta-review. Bmc Med Inform Decis. 8 10.1186/1472-6947-8-38 10.1186/1472-6947-8-38 PMC2551591 18789150

[38] Wagner AS, Milzer M, Schmidt ME, Kiermeier S, Maatouk I, Steindorf K. (2025) Nurses’ knowledge of cancer-related fatigue and the coverage of this subject in nursing training: a cross-sectional study. J Nurs Res. 33(2) ARTN e379 10.1097/jnr.0000000000000666 10.1097/jnr.0000000000000666 40162696

[39] Agbejule OA, Ekberg S, Hart NH, Chan RJ (2024) Supporting cancer-related fatigue self-management: a conversation analytic study of nurse counsellor and cancer survivor consultations. Eur J Oncol Nurs 73:102726. 10.1016/j.ejon.2024.102726 39522260 10.1016/j.ejon.2024.102726

[40] Dekker J, Sears SF, Åsenlöf P, Berry K (2023) Psychologically informed health care. Transl Behav Med 13(5):289–296. 10.1093/tbm/ibac105 36694354 10.1093/tbm/ibac105 PMC10182422

