# Supplementary material for: Toward a better understanding of the uptake of interventions for cancer-related fatigue: the perspective of healthcare providers, patients, and partners
Source: Support Care Cancer. 2025 Aug 1;33(8):740. doi: 10.1007/s00520-025-09765-3 (PMC12316727; doi:10.1007/s00520-025-09765-3)
Supplement: Supplementary file 1 — Supplementary file1 (DOCX 31.5 KB) [file 520_2025_9765_MOESM1_ESM.docx]

Supportive Care in Cancer

*Appendix 1 for the manuscript titled:*

Toward a better understanding of the uptake of interventions for cancer-related fatigue: the perspective of healthcare providers, patients and partners.

*Authors*

Kim F. Francken* ^1,2,3^, Annelotte Kooij^1^, Diana Zwahlen PhD^4,5^, Laurien M. Buffart PhD^6^, Joost Dekker PhD^2,3,7^, Hanneke W. M. van Laarhoven MD PhD^2,8^, Annemarie M. J. Braamse PhD^1,2,3^, Fabiola Müller PhD ^a,1,2,3^, Hans Knoop PhD^a,1,2,3^

^1^Amsterdam UMC location University of Amsterdam, Department of Medical Psychology, Amsterdam, The Netherlands

^2^Cancer Center Amsterdam, Cancer Treatment and Quality of Life, Amsterdam, The Netherlands

^3^Amsterdam Public Health, Mental Health, Amsterdam, The Netherlands

^4^Medical Oncology Department, University Hospital Basel, Basel, Switzerland

^5^Department of Psychosomatic Medicine, University Hospital Basel, Basel, Switzerland

^6^Department of Medical BioSciences, Radboud University Medical Center, Nijmegen, the Netherlands.

^7^Department of Psychiatry, Amsterdam UMC, Location Vrije Universiteit Amsterdam, Amsterdam, the Netherlands.

^8^Amsterdam UMC location University of Amsterdam, Department of Medical Oncology, Amsterdam, The Netherlands

*correspondence: k.f.francken@amsterdamumc.nl

^a^ Müller and Knoop contributed equally and share the last authorship

**Appendix 1. Interview questions**

- 1. **Interview guide patients**

**Introduction**

- Welcome the participant
- Introduction by interviewer
- Ask permission to record the interview
- Start recording

**Interview questions**

| Background | How are you doing today?  Can you tell me about current symptoms you are experiencing? |
| --- | --- |
| Fatigue | What is the influence of fatigue on your daily life?  How did your fatigue develop over time?  How do you feel about your fatigue? |
| Care Need | Do you / did you ever have a care need for fatigue?  Do you know what type of care for fatigue is available?  What are your expectations about care for fatigue? |
| Care Seeking | Did you ever seek care for fatigue?  Why did you / did you not seek care for fatigue?  How / where did you seek care? |
| Care Use | Did you ever use care for fatigue?  Why did you / did you not use care?  What type of care did you use / are you receiving?  How did you find this type of care? |
| Role healthcare practitioner | Did you ever talk about fatigue with your / a healthcare provider?  Did your healthcare provider give you advice about fatigue?  With which healthcare provider would you like to discuss fatigue? |
| Role partner | Do you discuss fatigue with your partner?  How does your partner deal with your fatigue?  Do you discuss care for fatigue with your partner?  Can you tell me about the role your partner played in whether or not to seek or use care? |

- 1. **Interview guide partners**

**Introduction**

- Welcome the participant
- Introduction by interviewer
- Ask permission to record the interview
- Start recording

**Interview questions**

| Background | Can you tell me something about the impact / consequences cancer has / had on your partner (i.e. the patient)? How do you notice this? |
| --- | --- |
| Fatigue experience | How do you notice fatigue in your partner (i.e. the patient)?  How did it start and how is it going now?  How do you deal with your partner’s fatigue?  Can you talk about it with your partner? |
| Fatigue impact | Did something change in your life because of fatigue?  How do you feel about that? |
| Care Need | Do you want your partner to get care for fatigue?  Do you think your partner has a need for care for fatigue? Why?  What do you know about care for fatigue? |
| Care Seeking | Did you ever look for information about care for fatigue? How?  Would you like to receive information about fatigue? How? When?  Do you know if your partner ever searched for care for fatigue?  Do you know what motivated them to seek care? |
| Care Use | Does your partner receive care for fatigue? Which care?  What was your partner’s main motivation to use care for fatigue?  Did you agree with this choice? |
| Role healthcare practitioner | Did you ever discuss care for fatigue with a healthcare practitioner?  With which healthcare provider would you like to discuss this? |

- 1. **Interview guide oncologists, nurses**

**Introduction**

- Welcome the participant
- Introduction by interviewer
- Ask permission to record the interview
- Start recording
- Round of introductions by participants

**Interview questions**

| Main questions | Do you ever discuss fatigue with your patients?   - Do you ask them about it? Does the patient bring it up? - Do you ask every patient? When do you ask? - How do you ask about it?   What do you know about the treatment options for fatigue? |
| --- | --- |
| Additional questions | What are your thoughts about fatigue after cancer?  Do you ever provide patients with advice regarding fatigue?   - What advice? - When? - Do you follow guidelines?   What determines if you refer someone to supportive care?   - And to what type of supportive care? - Do you experience any obstacles when you want to refer patients to supportive care?   What could be the benefits of discussing fatigue with patients?  Are there any reasons not to discuss fatigue with patients?  From your perspective, which patients want and/or seek care?  From your perspective, what are barriers for patients to seek/receive care? |

- 1. **Interview guide psychologists, physical therapists**

**Introduction**

- Welcome the participant
- Introduction by interviewer
- Ask permission to record the interview
- Start recording
- Round of introductions by participants

**Interview questions**

| Main questions | What are characteristics of the patients (with fatigue) you are treating?   - Which patients come to you for / seek help?   What are barriers you notice for patients to seek / use your type of treatment?   - Practical barriers? - Surrounding / social network? - Does everyone know where to find you? |
| --- | --- |
| Additional questions | How do patients end up being referred to your treatment facility/treatment type?   - At what timepoint? - Specifically for fatigue? Or usually for other symptoms? - Who refers patients to you?   What is the main reason patients end up being referred to your treatment facility/treatment type?   - What is their goal?   Is there a specific group that is not referred to you?   - Specific characteristics? - How is this possible? Can we solve that? |

- 1. **Interview guide general practitioners, social workers**

**Introduction**

- Welcome the participant
- Introduction by interviewer
- Ask permission to record the interview
- Start recording
- Round of introductions by participants

**Interview questions**

| Main questions | Do you ever discuss fatigue with your patients?   - Do you ask them about it? Does the patient bring it up? - Do you ask every patient? When do or don’t you? - How do you ask about it?   What advice do you give patients regarding fatigue?  What do you know about the treatment options for fatigue? |
| --- | --- |
| Additional questions | How do patients find you?   - At what timepoint? - Specifically for fatigue? Or usually for other symptoms? - Do other healthcare practitioners refer to you?   What determines if you refer someone to supportive care?   - And to what type of supportive care? - Do you experience any obstacles when you want to refer patients to supportive care?   Do you see benefits of discussing fatigue with patients?  From your perspective, which patients want and/or seek care?  From your perspective, what are barriers for patients to seek/receive care? |
